# Are Suicides Mad?

**Published:** 1894-11-10

**Authors:** 


### Studies in Applied Sociology.

### ARE SUICIDES MAD?

The burial penalty still attached to a verdict of *felo de se* is doubtless the chief reason why juries invariably declare that one who has killed himself "Committed suicide while suffering from temporary insanity." When the case is examined, we find often that "temporary insanity" is really the last explanation possible. "Domestic unhappiness" is a frequent cause; "deceased had been out of work for some time and was in low spirits" is another. Elsewhere than in England a sense of honour, misguided perhaps, but in no degree insane, often makes men take their own lives; and there have been periods in the world's history when suicide has been not merely honourable but fashionable, and men and women have ended their lives for reasons which to us seem trivial and absurd. These last bear a superficial, but only a superficial, resemblance to suicides based on insanity. In reality they are far apart from the instances of irrational but commanding impulse which can really be defined as suicidal mania.

When the mother of an illegitimate child, deserted by her lover and refused shelter in her own home, or permitted there only to be taunted incessantly with her fall—when she kills herself or her baby, or both, she is not mad. She is helpless, or, being ignorant, thinks herself so. She sees no way out of an intolerably humiliating position; she is miserable in the present and sees no hope in the future, therefore she ends her life. Her deed is, after a fashion, an active interpretation of the Psalmist's cry, "Let me fall into the hands of God, and not into the hands of man." It is foolish, cowardly, sinful; but it is not mad. Similarly, the man who has lost his occupation, and cannot bear to see his wife and children suffering privation, shows himself by his suicide to be selfish, cowardly, and lacking in foresight—for how will the loss of the possible breadwinner advantage those he loves?—but not, strictly speaking, mad, as madness is defined by alienists. Neither is the gambler who has lost his last penny, and blows out his brains at Monte Carlo, nor the ruined speculator, often fraudulent, who prefers death to poverty or prison. Where the average human mind can see a reason for despair, or even deep discouragement, we are not justified, in point of strict accuracy, in defining such suicide as due to "temporary insanity."

Are there then no cases where the verdict is true? Yes; but our sentimental leniency of condemnation makes it difficult to differentiate between them and these others we have spoken of. But it may be generally said that where outward circumstances give no explanation of the act, the suicide is one in which "temporary insanity" is the proper verdict. Suicide due to morbid philosophies—the "is life worth living?" theory, and the rest—occupy a middle place. Granted the premises of the philosopher, the path to suicide may be clearly enough defined; but it may reasonably be argued that the reasoning that leads to the act is so insufficient as to prove weakness of intellect, or at least a lack of truly philosophical breadth and insight, in the case of anyone who has adopted it. But leaving out these which each will judge according to his own prejudices, there remain many cases of suicide which madness only can explain.

Unheroic as the statement may seem, contempt of death is a virtue rather of a savage race. With civilisation comes greater susceptibility of nerve, which instinctively shrinks from pain. This shrinking may be overcome by the sense of duty, the instinct of affection, the hopes which religion inspires; we reach thus the highest heroism; but fundamentally the shrinking is there. When in civilised man or woman we meet with that unreasoning contempt of pain which leads to suicide, we may be sure that "that way madness lies." The madness may have shown itself in no other way; there may, indeed, have been exceptional mental activity, often success, and every sign of delight of life; yet, after all, the delight in life is less than the impulse to death. Occasionally the suicide leaves a letter avowing this irresistible impulse. How irresistible it is we realise best in those cases in which the first attempt is checked, only to be repeated on every possible opportunity, with endless ingenuity and persistence until, at last, in spite of all the watchers can do, the end is gained. The existence of this suicidal mania, like its kinsman, homicidal mania, is often known to those who suffer from it, and they voluntarily put themselves under restraint to be protected against themselves. Of course the mania is not always so pronounced as to find vent in action absolutely without cause, but when some very trivial vexation leads man or woman to take their own life, we may reasonably set them down as tainted with suicidal mania. Some time ago the newspapers chronicled the case of a woman who was quarrelling with a neighbour, and in her anger so lost self-control that she jumped out of a window and was killed. The act was instinctive, motiveless from a rational point of view, and one can only surmise that some latent suicidal impulse gained supreme control when her anger was aroused. Sometimes we hear of a girl committing suicide because she cannot go to a party of pleasure. But a hundred girls sustain the same disappointment without yielding to more than a burst of tears or a fit of sulking. The one who shows her vexation in so fatal a manner had, unsuspected by her nearest friends, a strong impulse to suicide, which wanted only the occasion to come to the surface. Of her fellows none will be so easily moved to self-destruction; but another may kill herself because of a disappointment in love, while a third will bear all the ills that life can bring without a thought of terminating them in such a fashion.

Whence arises this tendency? Sometimes we see a whole family who seem as naturally to take to self-murder as others do to drink; the morbid craving comes out in all, or nearly all, its members. But this is rare. Nevertheless it is rare for a suicide to come of a mentally healthy family. The aberration, the lack of balance, may show itself in a different form in different individuals, but generally the whole family shows the taint in one form or another. Search back the record, and you will probably find that one has been guilty of crime, another is epileptic, a third drunken, a fourth only harmlessly eccentric, but all showing, in one way or another, a difference from the ordinary level of that commonplace humanity which has no strong impulse either to good or ill.

Is the impulse to suicide curable? Not directly. It depends on family, on race, on the strain of that competition which marks our advancing civilisation. Of these the two first are ineradicable, though doubtless capable of being modified in the course of generations through judicious marriage. The third is, for the mass of men, unattainable; yet individuals who know that they have an hereditary taint might, of their own free will, withdraw from those occupations which rouse the nervous system to abnormal excitement, and, even at the sacrifice of some of the world's goods, lead wholesome lives, which would give the murderous instinct less chance to conquer them. Physical weakness, especially that resulting from over-strain, betrays and weakens the control over the mental flaw. 'Tis the old story: the *mens sana* cannot permanently dwell except in the *corpus sanum*.

